# A Spectroscopic Technique to Simultaneously Characterize Fatty Acid Uptake, Mitochondrial Activity, Vascularity, and Oxygen Saturation for Longitudinal Studies In Vivo

**DOI:** 10.3390/metabo12050369

**Published:** 2022-04-19

**Authors:** Riley J. Deutsch, Victoria W. D’Agostino, Enakshi D. Sunassee, Michelle Kwan, Megan C. Madonna, Gregory Palmer, Brian T. Crouch, Nimmi Ramanujam

**Affiliations:** 1 Department of Biomedical Engineering, Duke University, Durham, NC 27708, USA; rjd34@duke.edu (R.J.D.); enakshi.sunassee@duke.edu (E.D.S.); megan.madonna@duke.edu (M.C.M.); brian.crouch@duke.edu (B.T.C.); nimmi@duke.edu (N.R.); 2 Department of Biology, Duke University, Durham, NC 27708, USA; michelle.kwan@duke.edu; 3 Department of Radiation Oncology, Duke University, Durham, NC 27708, USA; greg.palmer@duke.edu; 4 Department of Pharmacology and Cancer Biology, Duke University Medical Center, Durham, NC 27708, USA

**Keywords:** optical spectroscopy, tumor metabolism, fatty acid uptake, murine tumor lines, mitochondrial metabolism, tumor vascular environment

## Abstract

Aggressive breast cancer has been shown to shift its metabolism towards increased lipid catabolism as the primary carbon source for oxidative phosphorylation. In this study, we present a technique to longitudinally monitor lipid metabolism and oxidative phosphorylation in pre-clinical tumor models to investigate the metabolic changes with mammary tissue development and characterize metabolic differences between primary murine breast cancer and normal mammary tissue. We used optical spectroscopy to measure the signal of two simultaneously injected exogenous fluorescent metabolic reporters: TMRE (oxidative phosphorylation surrogate) and Bodipy FL C16 (lipid catabolism surrogate). We leverage an inverse Monte Carlo algorithm to correct for aberrations resulting from tissue optical properties and to extract vascular endpoints relevant to oxidative metabolism, specifically oxygen saturation (SO_2_) and hemoglobin concentration ([Hb]). We extensively validated our optical method to demonstrate that our two fluorescent metabolic endpoints can be measured without chemical or optical crosstalk and that dual measurements of both fluorophores in vivo faithfully recapitulate the measurements of each fluorophore independently. We then applied our method to track the metabolism of growing 4T1 and 67NR breast tumors and aging mammary tissue, all highly metabolic tissue types. Our results show the changes in metabolism as a function of mammary age and tumor growth, and these changes can be best distinguished through the combination of endpoints measured with our system. Clustering analysis incorporating both Bodipy FL C16 and TMRE endpoints combined with either SO_2_ or [Hb] proved to be the most effective in minimizing intra-group variance and maximizing inter-group differences. Our platform can be extended to applications in which long-term metabolic flexibility is important to study, for example in tumor regression, recurrence following dormancy, and responses to cancer treatment.

## 1. Introduction

Dysregulated cellular metabolism is recognized as a core hallmark of cancer [1]. Tumor cells upregulate metabolic activity to sustain continuous proliferative growth, protect against damage from reactive oxygen species, sustain an acidic immunosuppressive environment, and generate cancer stem cells [2,3]. One of the foundational insights into cancer metabolism has been tumors’ propensity to increase the uptake of glucose and convert it into lactate via glycolysis rather than shuttling it into the Citric Acid Cycle and proceeding with oxidative phosphorylation [4]. As rapid proliferation outstrips angiogenesis, large portions of tumors exist in a hypoxic state. Subsequently, cancer cells upregulate aerobic glycolysis, diverting amino acids and lipids to biosynthetic processes [5]. Increased glycolysis is favorable as increased lactate production contributes to an acidified environment, promoting immune escape and decreasing mitochondrial activity [5]. Known as the Warburg effect, high rates of aerobic glycolysis have been exploited clinically for cancer diagnosis, staging, and monitoring (18F-deoxyglucose-positron emission tomography, FDG-PET).

Cancers are metabolically flexible and adaptive, often utilizing multiple metabolic pathways including oxidative phosphorylation, glycolysis, amino acid metabolism, the pentose phosphate pathway, and fatty acid β-oxidation [5,6]. Cancer cells can also reprogram their metabolism in response to metabolic stress from a changing tumor microenvironment, allowing them to escape treatment, lie dormant, and recur [7]. Metabolic reprogramming, including reliance on fatty acid β-oxidation and oxidative phosphorylation, is an underlying mechanism of chemo-resistance [8,9,10,11]. Dysregulated lipid metabolism is an important mechanism for tumor recurrence following therapy [12,13]. Triple Negative Breast Cancers (TNBC), a subtype with particularly poor clinical outcomes [14,15], show increased reliance on fats as an energy source compared to other subtypes of breast cancer [16]. Higher dependence on both oxidative phosphorylation and fatty acid β-oxidation has been demonstrated in Doxorubicin-resistant TNBC cells [17]. An increase in enzymes related to fatty acid β-oxidation has been documented in triple negative breast tumors of chemo-resistant patients [18]. Given that recent work has also pointed to an oncogene-driven dependence of fatty acid metabolism in certain other tumor types [14,19], methods allowing for the in vivo study of tumor lipid metabolism are pertinent for further metabolic studies.

To quantify the key metabolic features described above, we have previously shown that rhodamine derivative Tetra Methyl Rhodamine Ethyl ester (TMRE) and 4,4-Difluoro-5,7-Dimethyl-4-Bora-3a,4a-Diaza-*s*-Indacene-3-Hexadecanoic Acid (Bodipy FL C16) are two fluorescent indicators that report on mitochondrial membrane potential (oxidative phosphorylation surrogate) and fatty acid uptake (fatty acid β-oxidation surrogate), respectively. We and others showed TNBCs bearing the MYC oncogene have a high dependence on fatty acid β-oxidation and that the Bodipy FL C16 fluorescence signal can track changes in lipid metabolism following oncogene downregulation or chemical perturbation [14,20]. Further, we used TMRE in a separate study to image mitochondrial membrane potential (MMP) as an oxidative phosphorylation surrogate and a fluorescent glucose analog 2-(N-(7-nitrobenz-2-oxa-1,3-diazol-4-yl)amino)-2-deoxyglucose (2-NBDG) in an in vitro model of human epidermal growth factor receptor 2 (HER2)-positive breast cancer following HER2 oncogene withdrawal [21]. We showed that mammospheres expressing the HER2 oncogene have high glucose uptake and low MMP [21]. Upon oncogene downregulation, a significant decrease in glucose uptake and a significant increase in MMP persisted over the course of regression, dormancy, and recurrence [21].

Though these studies showed the significance of TMRE and Bodipy FL C16 as tools to characterize metabolic flexibility, they were measured in isolation. Given the inextricable link that lipid uptake and mitochondrial metabolism exhibit, we sought to develop an optical spectroscopy platform to simultaneously quantify the contributions of Bodipy FL C16 and TMRE as well as two endogenous endpoints relevant to oxidative phosphorylation and vascularization: oxygen saturation (SO_2_) and hemoglobin concentration ([Hb]). We demonstrate that all four endpoints can be measured simultaneously without chemical, optical, or biological crosstalk. We set out to demonstrate the utility of collecting two fluorescent metabolic endpoints simultaneously. We performed a principal component analysis followed by a graph-based clustering method to determine the metabolic dependence of different tissue phenotypes: mammary tissue by age and 4T1 and 67NR tumors (murine breast cancers lines). The analyses showed that a decrease in TMRE and [Hb] contributes to the clustering of older mammary groups (9–15 weeks of age) from their younger counterparts (7–8 weeks of age) in normal tissue, reflecting a decrease in energy needs with age. Bodipy FL C16, TMRE, and [Hb] all contribute to the clustering of 4T1 tumors from normal mammary, suggesting the importance of the lipid palmitate as a substrate. None of the four variables strongly described the clustering of 67NR tumors from corresponding normal mammary tissues in age-matched mice. Taken together, this study shows that our methodology can describe the metabolic dependence of different tissue phenotypes and longitudinally track these features in vivo. Our platform can be extended to applications in which long-term metabolic flexibility is important to study, for example in tumor regression and recurrence following dormancy, as well as responses to cancer treatment including metabolic inhibitors.

## 2. Results

### 2.1. There Is No Chemical Crosstalk between TMRE and Bodipy FL C16

**Figure 1:**
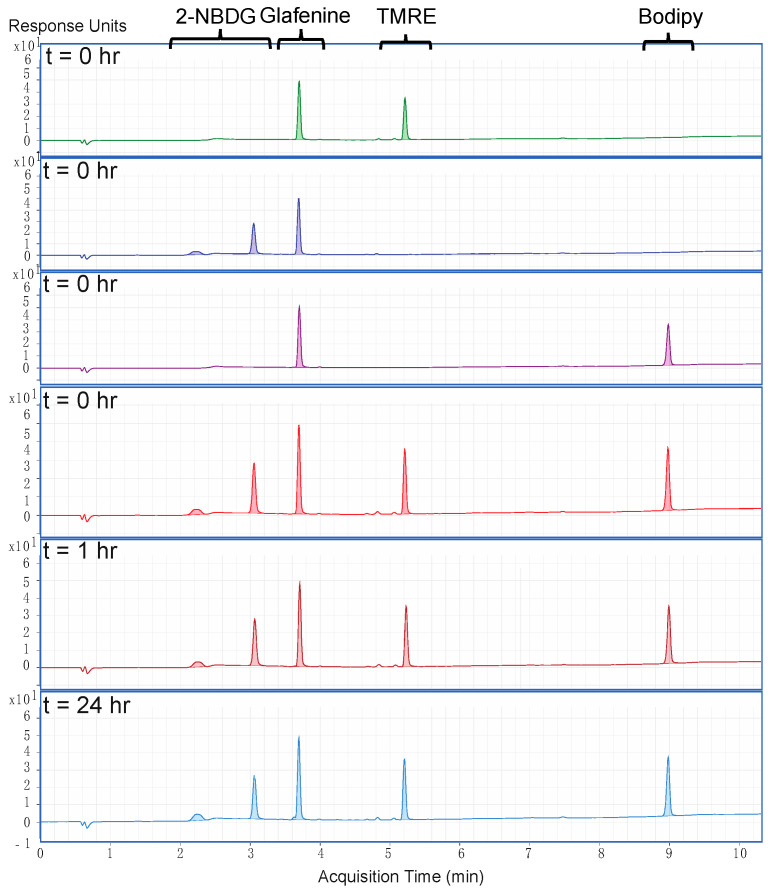
There is no chemical crosstalk between TMRE and Bodipy FL C16. Chromatograms (in descending order) of 100 mM TMRE, 2-NBDG, Bodipy FL C16, and a mixture of all three at t = 0, the mixture at t = 1 h, and the mixture at t = 24 h. All solutions contained Glafenine (20 μg/mL) as an internal standard with methanol as a solvent. The peak area was normalized to that of the internal standard. Change in peak area per compound was within 2% (within experimental variability), indicating no chemical change occurs after incubation of the compounds.

TMRE and Bodipy FL C16 do not chemically react when combined in solution; this was confirmed using a triple quadruple liquid chromatography mass spectrometer (LCMS-QqQ) to analyze individual and mixed solutions of TMRE and Bodipy FL C16. A third fluorescent metabolic probe, 2-NBDG, was included in the mixture as an individual compound and showed no chemical crosstalk with TMRE or Bodipy FL C16. Although not discussed further in this paper, 2-NBDG may be used in future metabolic studies. Figure 1 shows chromatograms of four solutions prepared with methanol as a solvent: (i) 100 μM TMRE, (ii) 100 μM 2-NBDG, (iii) 100 μM Bodipy FL C16, and (iv) a mixture of all three compounds each at 100 μM. All features from individual solutions were maintained upon mixing and incubation. Percent recovery of individual and mixed solutions, normalized to the peak area of the internal standard, demonstrate less than 2% variation (within experimental variability) at 0, 1, and 24 h after mixing, confirming that there is no chemical crosstalk between compounds.

### 2.2. The Fluorescence of TMRE and Bodipy FL C16 along with Optical Properties Can Be Extracted from Turbid Phantoms with No Optical Crosstalk

**Figure 2:**
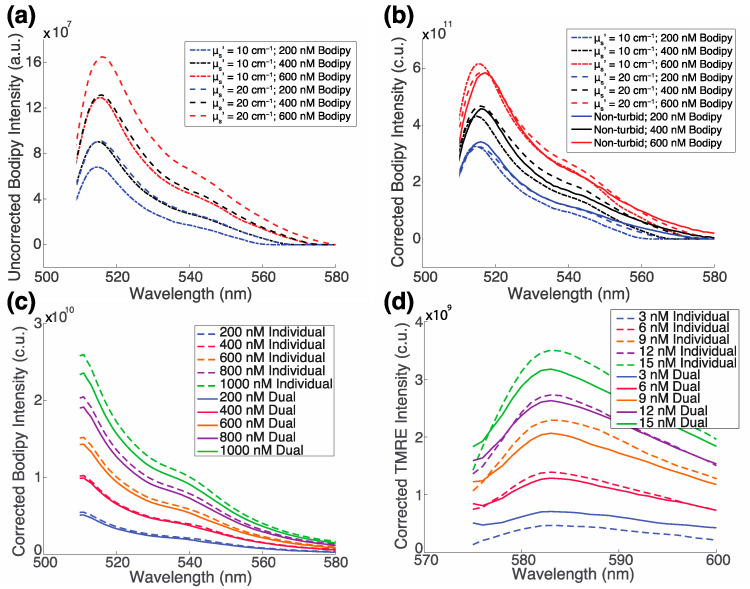
There is no optical crosstalk TMRE and Bodipy FL C16 in mixed samples. (**a**) Measured and (**b**) corrected fluorescence spectra at three Bodipy FL C16 concentrations, each with a fixed absorption coefficient (μ_a_) of 0.3 cm^−1^ and a high (20 cm^−1^) or low (10 cm^−1^) reduced scattering coefficient (μ_s_′). The Bodipy FL C16 concentrations are 200 nM, 400 nM, and 600 nM, respectively. (**c**) Corrected Bodipy FL C16 spectra with (solid lines) and without (dashed lines) 12 nM TMRE. Bodipy FL C16 concentrations are 200, 400, 600, 800, or 1000 nM and (**d**) corrected TMRE spectra with (solid lines) and without (dashed lines) 200 nM Bodipy FL C16. TMRE concentrations are 3, 6, 9, 12, or 15 nM. In each phantom in (**c**,**d**), the reduced scattering coefficient (μ_s_′) is 10 cm^−1^ and there is no absorber present. Intensity is reported in arbitrary units (a.u.) prior to correction and in corrected units (c.u.) after correction. The color indicates the same fluorophore concentration; dashed line = high scatter, dotted line = low scatter, and solid line = true fluorescence in a non-turbid solution. All fluorescence intensities are reported in corrected units (c.u.).

Figure 2 shows two sets of phantom experiments where one fluorophore concentration was varied while the other fluorophore concentration was fixed. Figure 2a,b shows the raw and inverse Monte Carlo corrected Bodipy FL C16 fluorescence spectra at concentrations of 200, 400, and 600 nM, with no TMRE, a fixed absorption coefficient (μ_a_) of 0.3 cm^−1^, and either a high (20 cm^−1^) or low (10 cm^−1^) reduced scattering coefficient (μ_s_′) in each. Figure 2c,d shows the inverse Monte Carlo corrected spectra of Bodipy FL C16 and TMRE, respectively, from solutions that have both metabolic indicators. The Bodipy FL C16 concentrations were 0, 200, 400, 600, 800, and 1000 nM. Each concentration was evaluated with or without a fixed concentration of 12 nM TMRE. The TMRE concentrations were 0, 3, 6, 9, 12, and 15 nM. Each concentration was evaluated with or without a fixed concentration of 200 nM Bodipy FL C16. Each phantom had a fixed absorption coefficient (μ_a_) of 0 cm^−1^ and a reduced scattering coefficient (μ_s_′) of 10 cm^−1^.

Upon examination of Figure 2a, it is evident that identical Bodipy FL C16 concentrations yield varying magnitudes of fluorescence emission intensities depending on the scattering properties of the phantom. Figure 2b compares the spectra corrected with the inverse Monte Carlo algorithm to true values from a non-turbid phantom (no scatterer). The corrected spectra have comparable magnitudes to that of the same fluorophore in a turbidity-free medium. Figure 2c shows that there is excellent concordance between varying concentrations of corrected Bodipy FL C16 with (solid line) and without 12 nM TMRE (dashed lines). Similar results are observed in the corrected TMRE spectra with (solid lines) and without 200 nM Bodipy FL C16 (dashed lines) as shown in Figure 2d.

**Figure 3:**
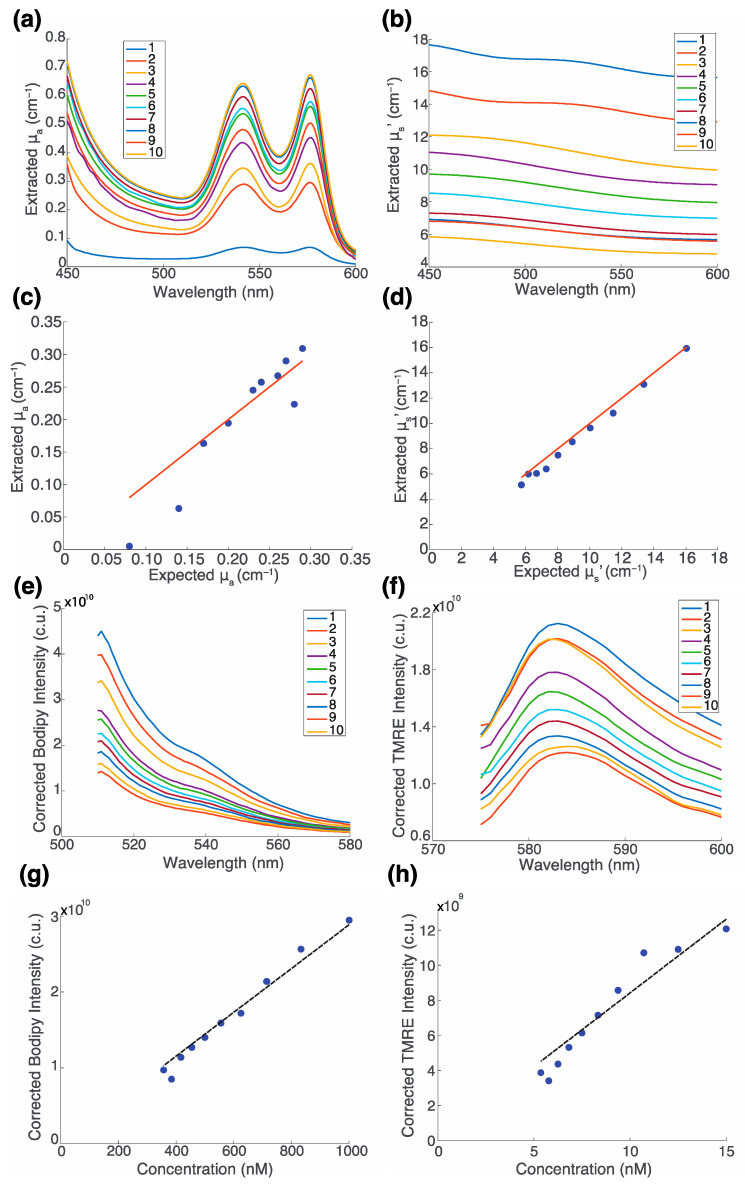
The fluorescence of TMRE and Bodipy FL C16 along with optical properties can be extracted from turbid phantoms. (**a**) Extracted absorption coefficient (μ_a_, cm^−1^) and (**b**) extracted reduced scattering coefficient (μ_s_′, cm^−1^) across wavelengths 450 nm to 600 nm for each of ten phantoms. (**c**) Expected vs. extracted absorption coefficient (μ_a_, cm^−1^, R^2^ = 0.9116); (**d**) Expected vs. extracted reduced scattering coefficient (μ_s_′, cm^−1^, R^2^ = 0.9964); the red line represents y = x. Inverted (**e**) Bodipy FL C16 and (**f**) TMRE spectra for all ten phantoms. Extracted fluorescence signal vs. concentration for (**g**) Bodipy FL C16 (R^2^ = 0.9721) and (**h**) TMRE (R^2^ = 0.9183). Points in (**c**,**d**,**g**,**h**) represent single measurements. The dashed black line in (**g**,**h**) represents the line of best fit passing through the origin (0,0). Intensity is reported in corrected units (c.u.).

A second phantom study was performed with phantoms composed of variable concentrations of hemoglobin, polystyrene spheres, TMRE, and Bodipy FL C16 to reflect the variable contributions of all four components. Table 1 shows the composition of each phantom. Figure 3a,b shows the extracted μ_a_ and μ_s_′ spectra, respectively, for each of the ten phantoms. Figure 3c shows the extracted vs. expected μ_a_ (R^2^ = 0.9116). Figure 3d shows the extracted vs. expected μ_s_′ (R^2^ = 0.9964). The deviation in the results of the measured μ_a_ for the lowest two values is likely due to the low concentration of absorber in the phantom. Figure 3e,f show the corrected Bodipy FL C16 and TMRE spectra, respectively, for all ten phantoms. Figure 3g,h show the corrected fluorescence for Bodipy FL C16 and TMRE, respectively. Bodipy FL C16 (Ex: 488 nm, Em: 510 nm) and TMRE (Ex: 555 nm, Em: 585 nm) intensity were measured and reported as the average intensity of wavelengths within 2.5 nm of the emission peak. Fluorophore concentrations were chosen to be biologically relevant based on empirical measurements reported previously [16,22]. As expected, both Bodipy FL C16 and TMRE fluorescence increase linearly as the concentration of fluorophore increases (R^2^ = 0.9721and R^2^ = 0.9183, respectively). The solid red line in Figure 3c,d is y = x. The dashed black line in Figure 3g,h is the line of best fit for concentration vs. intensity passing through the origin (0,0).

### 2.3. There Is No Biological Crosstalk between TMRE and Bodipy FL C16

**Figure 4:**
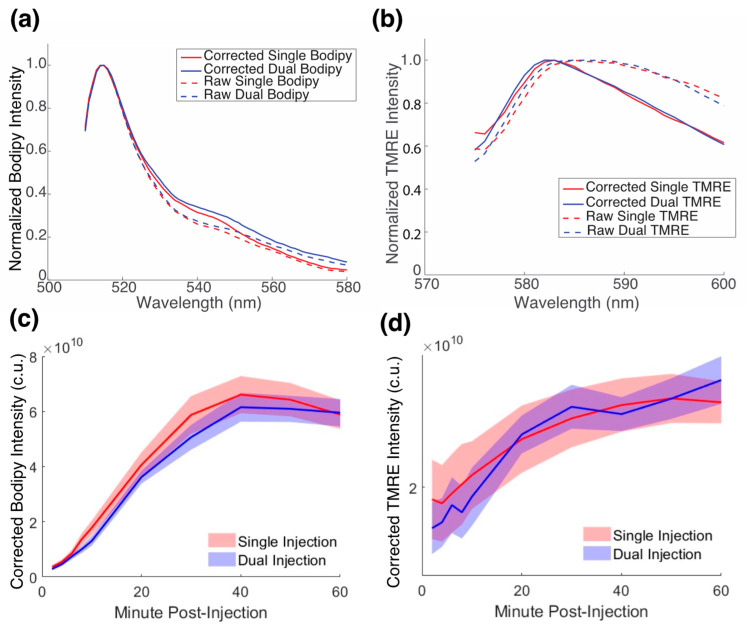
There is no biological crosstalk between TMRE and Bodipy FL C16. 4T1 mammary tumors were orthotopically injected into the fourth mammary fat pad in four cohorts of *n* = 9 Balb/c mice for the experimental studies. (**a**) Bodipy FL C16 and (**b**) TMRE spectra were measured and corrected with an inverse Monte Carlo algorithm and normalized to peak intensity for both individual and dual injections. Representative spectra are shown. (**c**) Single 200 μM injections of Bodipy FL C16 and dual injections of 200 μM Bodipy FL C16 and 75 μM TMRE over a 60-min period, post-injection, and (**d**) a single 75 μM injection of TMRE and dual injections of 200 μM Bodipy FL C16 and 75 μM TMRE for a 60 min period, post-injection. Intensities were averaged over nine animals, at each time point, and the shaded region represents standard (SE). *p* = n.s. for both comparisons. Intensity is reported in corrected units (c.u.).

Having demonstrated that there is neither optical nor chemical crosstalk between TMRE and Bodipy FL C16, we next aimed to show that neither compound interfered biologically with the other in vivo. Three cohorts of *n* = 9 BALB/c mice were injected retro-orbitally with either individual TMRE, individual Bodipy FL C16, or a dual injection of the two for in vivo spectroscopy. Figure 4a,b shows both measured and corrected representative spectra of Bodipy FL C16 and TMRE, respectively. The results show that there is excellent agreement between the dual probe and single probe measurements for each fluorophore. While correction minimally impacts Bodipy FL C16 fluorescence, the corrected TMRE fluorescence peak is left-shifted and corresponds to the actual peak of the TMRE fluorophore [22]. Figure 4c,d show corrected Bodipy FL C16 and TMRE fluorescence, respectively, at their peak wavelengths (Bodipy FL C16: 510 nm and TMRE: 585 nm), over a period of 60 min post-injection, averaged over nine animals at each timepoint. The shaded regions represent standard error. Dual and individual delivery curves for each fluorophore were tested for significance by repeated measures ANOVA. The delivery curves of a single injection were not significantly different from that of the dual injection for both TMRE and Bodipy FL C16.

### 2.4. Bodipy FL C16-TMRE Spectroscopy Measurements Reveal Metabolic and Vascular Changes in Normal Mammary Glands throughout Development

**Figure 5:**
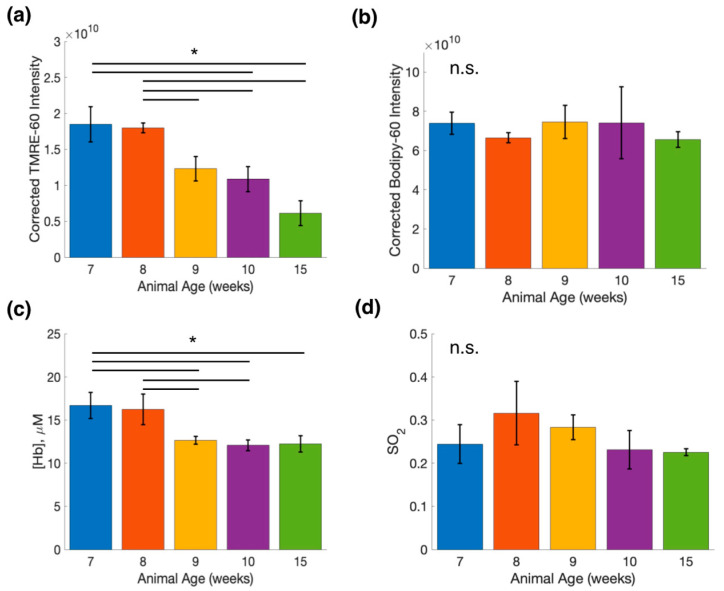
Bodipy FL C16-TMRE spectroscopy measurements reveal metabolic and vascular changes in normal mammary glands throughout development. (**a**) Comparison of mean TMRE fluorescence intensity for mammary tissue at different ages. (**b**) Comparison of mean Bodipy FL C16 fluorescence intensity for mammary tissue at different ages. (**c**) Comparison of total hemoglobin concentration ([Hb]) for mammary tissue at different ages. (**d**) Comparison of oxygen saturation ([SO_2_]) for mammary tissue at different ages. Error bars represent the standard error (SE) of the mean along each axis. For each timepoint *n* = 5, except for weeks 8 and 15 where *n* = 4. Statistical analysis was performed using a Wilcoxon rank-sum test to compare means. * Is *p* < 0.05 for all comparisons. n.s. is not significant.

The bar graph in Figure 5a shows the relationship between average TMRE intensity and animal age. TMRE intensity at weeks 10 and 15 are significantly decreased from weeks 7 and 8. Further, TMRE intensity significantly decreases from weeks 8 to 9. On the other hand, the bar graph in Figure 5b shows there is no significant change in average Bodipy FL C16 intensity between any of the groups. Similar to that observed in Figure 5a,c, a significant decrease in [Hb] in older compared to younger mammary tissue is shown. Specifically, [Hb] at weeks 9, 10, and 15 are significantly different from week 7, and weeks 9 and 10 are significantly different from week 8. As shown in Figure 5d, no statistically significant change was observed in SO_2_. Together, these data reflect age-related changes in metabolism and vascular endpoints of normal mammary tissue.

**Figure 6:**
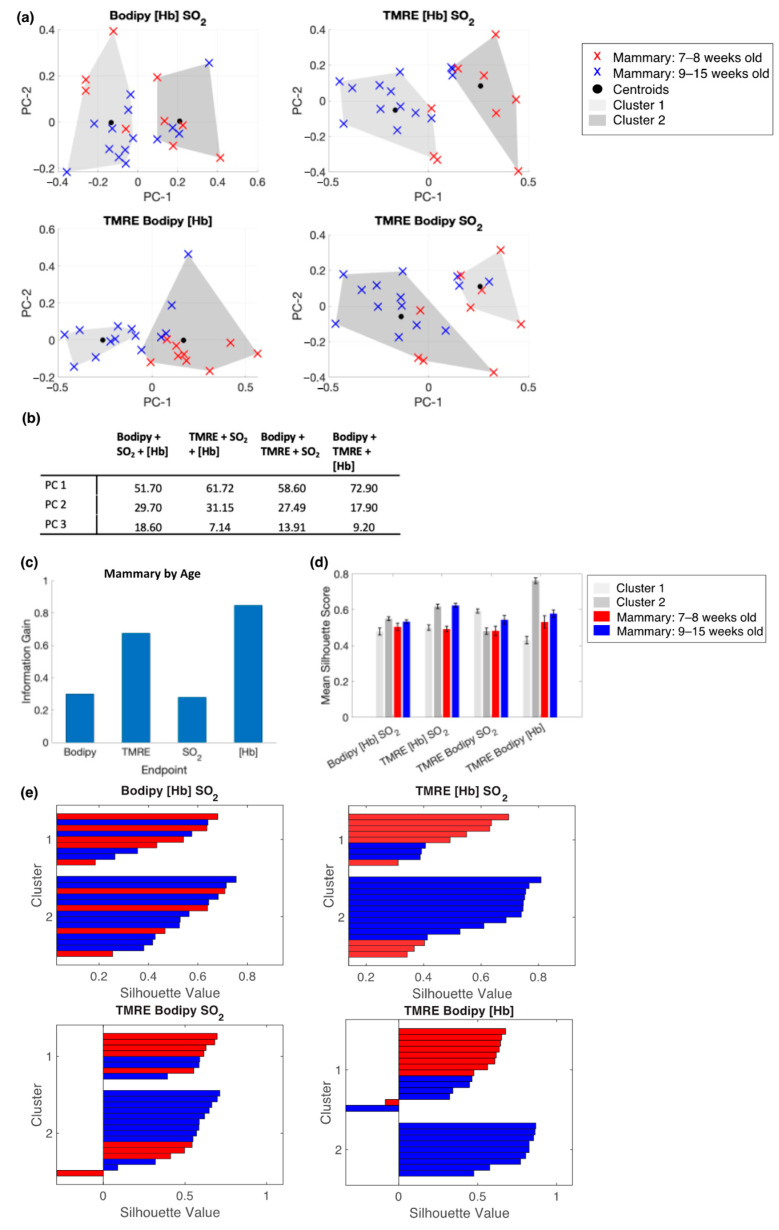
Bodipy FL C16-TMRE spectroscopy measurements reveal longitudinal changes in normal mammary glands throughout development. (**a**) Projections of the first two PCs show spectral clustering results for four different combinations of the following endpoints: Bodipy FL C16 fluorescence intensity, TMRE fluorescence intensity, total hemoglobin concentration ([Hb]), and total oxygen saturation (SO_2_). Fluorescence intensity is reported as the average intensity within 2.5 nm of the emission peak. (**b**) Table containing the variance described by each PC, by endpoint combination. (**c**) The calculated information gained from each variable. (**d**) The mean silhouette scores for cluster 1, cluster 2, data points with a true label of 7–8-week-old mammary, and data points with a true label of 9–15 week-old mammary. Error bars are SE. (**e**) Silhouette plots for each endpoint combination.

**Figure 7:**
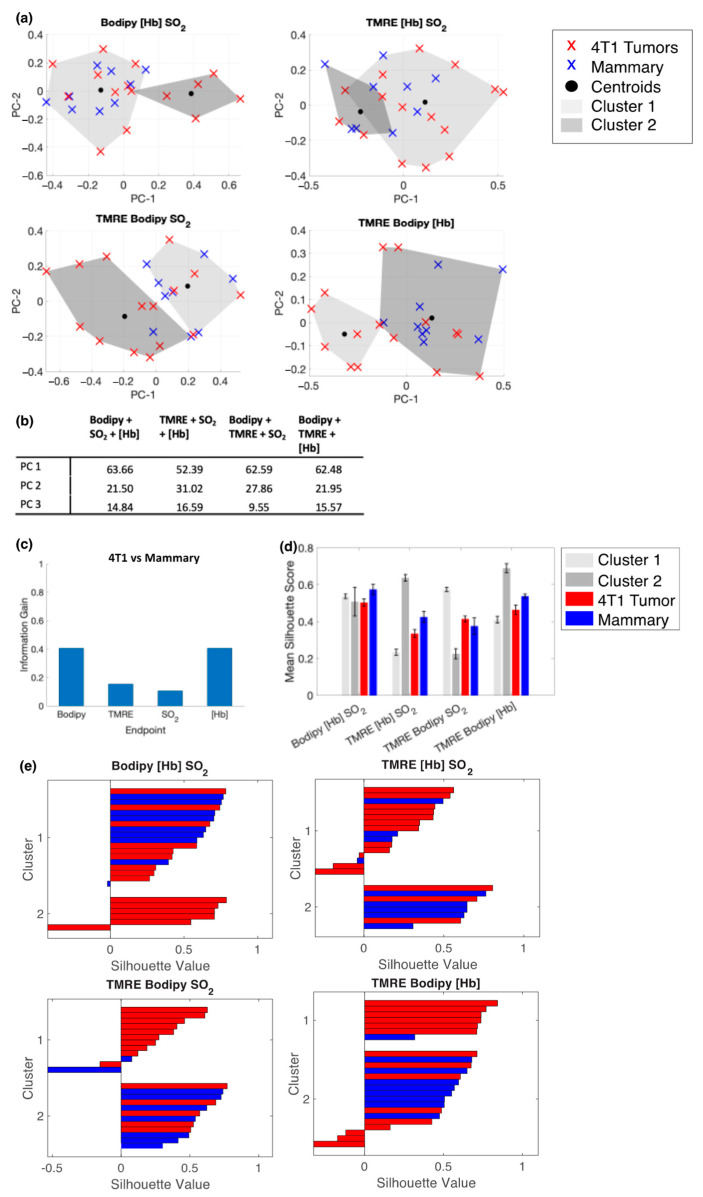
Bodipy FL C16 and TMRE fluorescence measurements combined with extracted vascular parameters separate clusters of normal and 4T1 tumor tissue. (**a**) Projections of the first two PCs show spectral clustering results for four different combinations of the following endpoints: Bodipy FL C16 fluorescence intensity, TMRE fluorescence intensity, total hemoglobin concentration ([Hb]), and total oxygen saturation (SO_2_). Fluorescence intensity is reported as the average intensity within 2.5 nm of the emission peak. (**b**) Table containing the variance described by each PC, by endpoint combination. (**c**) The calculated information gained from each variable. (**d**) The mean silhouette scores for cluster 1, cluster 2, data points with a true label of 4T1 tumors, and data points with a true label of healthy mammary tissue. Error bars are SE. (**e**) Silhouette plots for each endpoint combination.

**Figure 8:**
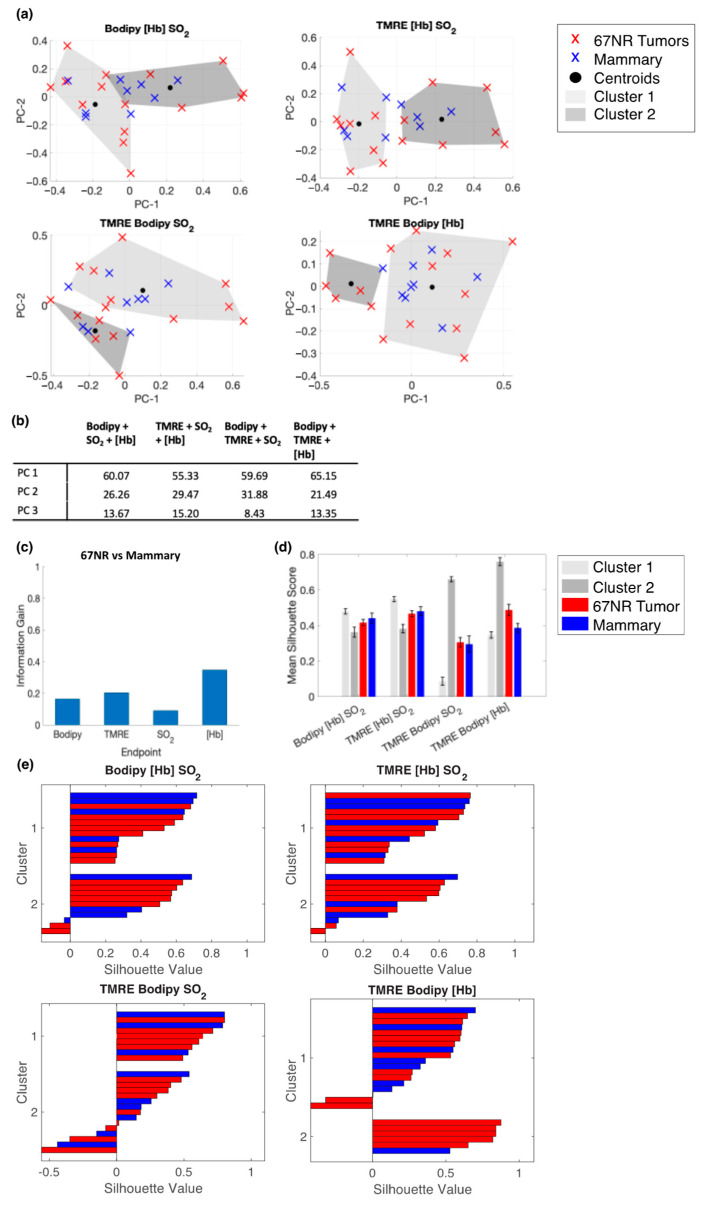
Bodipy FL C16 and TMRE fluorescence measurements combined with extracted vascular parameters do not separate clusters of normal and 67NR tumor tissue. (**a**) Projections of the first two PCs show spectral clustering results for four different combinations of the following endpoints: Bodipy FL C16 fluorescence intensity, TMRE fluorescence intensity, total hemoglobin concentration ([Hb]), and total oxygen saturation (SO_2_). Fluorescence intensity is reported as the average intensity within 2.5 nm of the emission peak. (**b**) Table containing the variance described by each PC, by endpoint combination. (**c**) The calculated information gained from each variable. (**d**) The mean silhouette scores for cluster 1, cluster 2, data points with a true label of 67NR tumors, and data points with a true label of healthy mammary tissue. Error bars are SE. (**e**) Silhouette plots for each endpoint combination.

We performed principal component analysis (PCA) on each group of interest separately (Mammary by age, 4T1 vs. mammary, and 67NR vs. mammary) and the results are shown in Figure 6, Figure 7 and Figure 8. We then applied spectral clustering to the PCs describing combinations of three of our four endpoints ((i) TMRE, SO_2_, [Hb]; (ii) Bodipy FL C16, SO_2_, [Hb]; (iii) TMRE, Bodipy FL C16, [Hb]; (iv) TMRE, Bodipy FL C16, SO_2_). Figure 6a shows the results of spectral clustering using all three PCs (total variance = 100%). Plots are projections of the first two PCs. Figure 6b shows the variance in each PC. To characterize how well each variable described our data, we performed information gain calculations for each endpoint on three different subsets of data. Figure 6c shows the results of the information gain calculations for our longitudinal mammary data. As expected, most information gain lies in TMRE and [Hb]. We then performed spectral clustering to demonstrate how two fluorescent metabolic measurements better describe the dataset compared to a single measurement. To quantify the results of clustering, we calculated a silhouette score for each data point to report how well that point fits in its assigned cluster. Silhouette scores measure how similar a data point in a cluster is to its own cluster (cohesion) compared to other clusters (separation). Silhouette scores range from −1 to 1, with a higher score indicating that clusters are well distinguished. Clusters with a score of 0.5 or higher are considered well separated. Figure 6d shows the average silhouette scores by cluster and by tissue type. Here, all average silhouette scores are comparable, pointing to the ease of separation of this dataset. The silhouette plots shown in Figure 6e, demonstrate that the clustering analyses using TMRE and [Hb] most consistently separate data points into young (week 7–8) or old (week 9–15) clusters. Conversely, clustering using Bodipy FL C16, [Hb], and SO_2_ results in clusters containing data points from each age (young or old) with high silhouette scores in each cluster.

### 2.5. Bodipy FL C16 and TMRE Fluorescence Measurements Combined with Extracted Vasculature Parameters Show the Clustering of Tumors and Mammary Tissues in Age Matched Mice

We next attempted to cluster tumor and normal tissue, specifically, 4T1 measurements and mammary measurements from age-matched mice at weeks 7 and 8; and 67NR measurements and mammary measurements from age-matched mice at weeks 7 and 8. As shown in Supplementary Figure S1, there is no statistically significant difference observed between any groups. Inverse Monte Carlo corrected fluorescence spectra for Bodipy FL C16 and TMRE at the 60 min post-injection timepoint for mammary tissue, 4T1 tumors, and 67NR tumors are shown in Supplementary Figures S2–S4, respectively.

We performed PCA on these groups using all combinations of three of our four endpoints ((i) TMRE, SO_2_, [Hb]; (ii) Bodipy FL C16, SO_2_, [Hb]; (iii) TMRE, Bodipy FL C16, [Hb]; (iv) TMRE, Bodipy FL C16, SO_2_). The first three PCs for the 4T1 vs. mammary group and 67NR tumors vs. the mammary group were used for the clustering analysis (Figure 7 and Figure 8).

Figure 7a shows the clustering of the first two PCs from different combinations of metabolic and vascular inputs; plots are projections of the first two PCs. Figure 7b contains the variance described by each PC. For 4T1 tumors, Bodipy FL C16 and [Hb], and to a lesser extent TMRE, have the highest information gain, shown in Figure 7c. The average silhouette scores for each cluster and each tissue type are shown in Figure 7d. Here, clusters generated from TMRE, Bodipy FL C16, and [Hb] produce higher mean silhouette scores than the other endpoint combinations. From the individual silhouette scores (Figure 7e), we see that the clusters formed based on this set of endpoints are more effective at separating the 4T1 tumors from normal mammary tissue types.

We performed the same analyses to differentiate mammary (week 7–8) and 67NR tumor data. Figure 8a shows the clustering results of the PCs for each subset of metabolic endpoints. Plots are projections of the first two PCs. Figure 8b contains the variance described by each PC. Figure 8c shows that for 67NR tumors vs. mammary tissue, [Hb] and TMRE have the highest information gain, similar to that in normal mammary tissues from 7–8 weeks of age, which is likely why there are no discernible differences between 67NR tumors and mammary tissues. Figure 8d shows average silhouette scores for each subset of metabolic endpoints. Figure 8e shows that silhouette scores generated with Bodipy FL C16, TMRE, and SO_2_ produced multiple negative silhouette scores, suggesting a lower separation in the group that lacked [Hb] information. In total, we further illustrate the value of having multiple metabolic endpoints and the added benefit of extracting vascular properties from diffuse reflectance data.

## 3. Discussion

Measuring multiple metabolic endpoints from a single tissue sample provides a more holistic understanding of how metabolic pathways change over time. We demonstrate that both our fluorescent and vascular endpoints all provide information to describe the data depending on the context. Although different combinations of three of the four endpoints could be used, incorporating both Bodipy FL C16 and TMRE endpoints in any combination of three proved to be the most effective in minimizing intra-group variance and maximizing inter-group differences. We saw that collectively analyzing multiple endpoints effectively delineated clusters of normal mammary and tumor tissues. These observations underscore the importance of examining the relationship between the metabolic and vascular variables within a tissue or tumor group rather than using individual endpoints that may by themselves not fully describe these features.

Others have also shown that increasing the number of optical endpoints allows for a better classification of tumors. Autofluorescence metabolic imaging, stimulated Raman scattering (SRS), Coherent anti-Stokes Raman scattering (CARS) imaging, and spectroscopy utilize multiple laser sources to report on key biomolecules and pathways in tissue such as lipid uptake [23], protein metabolism [24], and glycolysis [25]. For instance, previous groups have combined information related to the optical redox ratio (quantification of FAD and NADH fluorescence) and fluorescence lifetime to identify metabolic trends in cultured cells experiencing perturbations such as hypoxia and hypothermia [26]. Similar to our technique, redox imaging can draw conclusions about the use of oxidative phosphorylation compared to other metabolic pathways. Others have shown the value of using multiple Raman peaks to measure a large number of biomolecules (lipids, nucleic acids, and collagen) to better categorize radiation resistant tumor lines [27]. In each of these cases, the inclusion of multiple metabolic endpoints enabled higher dimensional analyses with clustering or classification tools.

Our study also provided insight into how the age of mammary tissue affects the interpretation of studies comparing tumor and healthy mammary tissue. From approximately 3 weeks of age to approximately 10–12 weeks of age, the mammary gland undergoes rapid development including infiltration and elongation of the ductal tree and adipocyte development near ductal structures [28,29]. This should lead to a change in metabolism as a function of energy demands from cellular proliferation. Our longitudinal fluorescence spectroscopy measurements revealed a statistically significant decrease in TMRE uptake in animals aged 9–15 weeks compared to those at 7–8 weeks of age. We also observed a decrease in [Hb] over this period. Interestingly this was not observed for either Bodipy FL C16 or SO_2_. Though studying the molecular mechanisms of these changes is beyond the scope of this work, these results underscore the importance of studying metabolism in age-matched controls and tumors.

Multi-parametric metabolic measurements improve the ability to distinguish tissue types. Previously, we have not observed statistically significant differences in TMRE or Bodipy uptake between 67NR and 4T1 tumor lines [16,30,31] using our optical toolbox. The findings in this study match previous work. While it is possible to observe differences in fluorescence signal between tumors and healthy dorsal skin or flank muscle, we observed here that normal developing mammary tissue exhibits similar levels of fatty acid uptake and mitochondrial membrane potential compared to a tumor. By simultaneously analyzing our four metabolic endpoints, it is possible to better differentiate tumor and normal mammary tissue types. Information gain analysis of data from normal mammary tissue and 4T1 and 67NR tumor tissue reveals that [Hb] and, for 4T1 tumors, Bodipy FL c16 are important to describing our tumor tissue. Clustering analysis supports this conclusion by separating tumor and normal tissue with combinations of [Hb] and metabolic endpoints.

Understanding the importance of glucose as a substrate for cancer metabolism is important and it would be beneficial to have a method to collect longitudinal measurements of all three endpoints in vivo. Our group has previously validated the use of glucose analog 2-(N-(7-nitrobenz-2-oxa-1,3-diazol-4-yl)amino)-2-deoxyglucose (2-NBDG) with TMRE to report on glucose uptake and mitochondrial membrane potential to provide insight into tumor reliance on glycolysis or oxidative phosphorylation or both for fuel [22,31,32]. Further, we have developed a method to measure 2-NBDG and TMRE simultaneously in a single pre-clinical tumor [32] and used it to study relationships between the uptake of the two probes for tumors of different metastatic potential and at different stages of growth [22,31,32]. Therefore, future optical studies similar to those described in this paper to minimize optical and biological crosstalk between these three indicators should lead to a versatile multi-parametric platform for tissue metabolic spectroscopy well suited to pinpoint metabolic dependence and plasticity of cancer. These measurements can inform on windows for in-depth tissue analysis, using assays such as next generation sequencing or omic-based methods to quantify entire metabolic pathways and identify molecular mechanisms of disease progression.

We have developed an optical toolkit to characterize major relevant axes of cancer metabolism longitudinally in vivo. Simultaneous spectroscopy of TMRE and Bodipy FL 16 along with vascular endpoints is well poised to provide insight into metabolic plasticity and, therefore, can be readily applied to longitudinal studies of treatment resistance and recurrence—two outstanding challenges in cancer therapy [33]. With work pointing to the increased role of lipid metabolism in residual tumor cells [13] and the plasticity of mitochondrial function throughout tumor progression [34], simultaneous, longitudinal measurements of these two critical endpoints will allow for a more complete understanding of the interplay of these two processes.

## 4. Materials and Methods

### 4.1. Ethics Statement

All animal work was carried out in accordance with the recommendations in the Guide for the Care and Use of Laboratory Animals of the National Institutes of Health. The protocol was approved by the Duke University Institutional Animal Care and Use Committee (protocol number A038-21-02). All experiments were performed under isoflurane gas anesthesia, and all efforts were made to minimize suffering.

### 4.2. Liquid Chromatography-Mass Spectrometry of Fluorophore Samples

Quantitative liquid chromatography-mass spectrometry (LCMS) was performed on samples of 2-NBDG, TMRE, and Bodipy FL C16 with Glafenine as an internal standard to analyze fluorophore stability and to allow for quantitative analysis. All metabolic compounds were dissolved in dimethyl sulfoxide (DMSO) and diluted in methanol. Four solutions were prepared: (i) 100 μM 2-NBDG, 20 μM Glafenine; (ii) 100 μM TMRE, 20 μM Glafenine; (iii) 100 μM Bodipy Fl C16, 20 μM Glafenine; and (iv) 100 μM 2-NBDG, 100 μM TMRE, 100 μM Bodipy FL C16, and 20 μM Glafenine. The concentration was chosen to be a greater concentration than would be used experimentally for animal studies such that any possibility for chemical reactions between compounds would be captured by the system’s dynamic range. All solutions were analyzed immediately upon preparation and after 1 and 24 h. LCMS analysis was performed using an Agilent 6460 Triple Quadrupole LC-MS unit (Agilent Technologies, Santa Clara, CA, USA) and the ultraviolet/visible (UV-vis) absorption spectrum was measured at 345 nm. Chromatography was performed on a Phenomenenx Luna C18 column, 2 mm × 100 mm, 3 μ particle, with 5 μL injection volume, using solvents A: 100:3:0.3 water:MeOH:formic acid and B: 100:3:0.3 MeCN:water:formic acid. The gradient separation method was 0–100% B over 9 min, with a flow rate of 0.5 mL/min. All UV absorption peaks related to fluorophores and Glafenine were manually integrated and normalized to the peak area of Glafenine to determine percent recovery. Peak area was compared between individual solutions (i), (ii), (iii), and mixed solution (iv) at 0, 1, and 24 h to determine percent change.

### 4.3. Optical Measurements

Optical measurements for all studies were collected using a previously developed, validated, and reported optical spectroscopy system and optical fiber-based probe [22,35]. Briefly, the system consists of a 450 W Xenon lamp, a monochromator, a spectrograph, and a 2D CCD camera (Jobin Yvon Horiba, Edison, NJ, USA). The probe consists of 19 fibers for excitation illumination and 18 for emission detection (RoMack Inc., Irving, TX, USA). Each fiber had a numerical aperture of 0.22 and the sensing depth of the system has previously been estimated to be 1.5 mm. To account for day-to-day variations in the system, reflectance and fluorescence spectra were calibrated to a 20% reflectance standard (Spectralon, Labsphere) and a fluorescence standard (USF 210-010, Labsphere Inc., North Sutton, NH, USA), respectively. Diffuse reflectance measurements were collected by illuminating the tissue with broadband white light and scanning the emission monochromators across the wavelength range of interest (350–700 nm). Fluorescence measurements were collected by fixing the source monochromator to provide the required excitation wavelength while scanning the detection monochromator over the desired spectral range. The intensity calibrated fluorescence spectra were also scaled wavelength-by-wavelength with a correction factor determined by the fluorescence spectrum of a NIST-approved tungsten calibration lamp (Optronic Laboratories Inc., Orlando, FL, USA).

All measurements were acquired in a dark room. Optical spectroscopy measurements on both the phantoms and animal models were conducted after adequate time was allowed for system warm-up (>30 min). Reflectance spectra were acquired from 420–760 nm (acquisition time: 0.025 s); Bodipy FL C16 fluorescence spectra were acquired from 505–635 nm (acquisition time: 2 s) using excitation at 488 nm; TMRE fluorescence spectra were acquired from 575–705 nm (acquisition time: 5 s) using excitation at 555 nm. Background spectra were collected for both phantoms and animals before the addition of fluorophore.

### 4.4. Inverse MC Models for Reflectance and Fluorescence

The measured reflectance and system response calibrated fluorescence spectra for both phantom and pre-clinical studies were input into a scalable inverse Monte Carlo model. Previous work has validated the ability of this model to extract optical properties and intrinsic fluorescence signals from both liquid phantoms and tissue [36,37,38,39]. To correct the measured fluorescence spectrum for effects of tissue optical properties, it is necessary to know the optical absorption and scattering properties of the medium. The inverse Monte Carlo first extracts the absorption coefficient and reduced scattering coefficient from a diffuse reflectance spectrum. These extracted optical properties are then used by the inverse Monte Carlo fluorescence model to correct for the effects of scattering and absorption on the measured fluorescence spectrum to provide the intrinsic fluorescence. A measured “reference” phantom with known optical properties is used to generate the calibration factor to appropriately scale the outputs of the inverse Monte Carlo algorithm. The reference phantom was selected to minimize the error of extraction both absorption and scattering as previously described [36].

### 4.5. Tissue Phantoms

Three sets of liquid phantoms were created to demonstrate (i) the inverse Monte Carlo algorithm can reliably extract the intrinsic Bodipy FL C16 signal from phantoms with different optical properties, (ii) Bodipy FL C16 and TMRE exhibit no optical crosstalk when combined in solution, and (iii) differing levels of Bodipy FL C16, TMRE, and tissue optical properties can be reliably extracted from optical spectra using the inverse Monte Carlo algorithm. Liquid phantoms with tissue-mimicking properties were prepared using varying absorber, scatterer, and fluorophore concentrations. Hemoglobin (H0267, Sigma-Aldrich Co., St. Louis, MO, USA) was used as the non-fluorescent absorber, and 1-μm monodisperse polystyrene spheres (1-μm diameter, Catalog No. 07310, Polysciences, Warrington, PA, USA) were used as the scatterer. Mixing known volumes of stock hemoglobin and microsphere suspensions in deionized (DI) water with stock fluorophore solution allowed accurate control of the final absorption, scattering, and fluorescence properties in each phantom. The absorption spectra of the stock hemoglobin were measured using a spectrophotometer (Cary 300, Varian, Inc., Palo Alto, CA, USA) and were used to determine the final absorption of the phantom, while the values of the reduced scattering coefficients in the phantoms were calculated from the Mie theory for spherical particles using freely available software [40]. Bodipy FL C16 dissolved in DMSO exhibited a previously characterized red-shift [41] in the emission spectrum. Therefore, Bodipy FL C16 was diluted in 0.02 g/mL bovine serum albumin (Life Technologies/Thermo Fisher Scientific, Waltham, MA, USA) in PBS, with an expected protein-bound emission peak of 510 nm. Reflectance and fluorescence measurements from the phantoms were obtained by placing the optical probe just beneath the surface of the liquid with the phantom being well mixed by repeated pipetting before each measurement.

To demonstrate the ability of the inverse Monte Carlo algorithm [38,39] to correct for the effects of tissue optical properties (scattering and absorption) of the measured Bodipy FL C16 spectra, we constructed three pairs of liquid phantoms, with each pair containing a fixed concentration of fluorophore and both a high (m_s_’ = 20 cm^−1^) and low (m_s_’ = 10 cm^−1^) concentration of scatterer. Phantom pairs contained Bodipy FL C16 at 200 nM, 400 nM, and 600 nM. Concentrations are based on biologically relevant fluorophore concentrations in previous in vivo microscopy studies [16]. To appropriately compare non-turbid and turbid phantoms, a scaling factor was applied to all non-turbid spectra based on a ratio of the peak intensity of the phantom containing Bodipy FL C16 at 200 nM, low-scatter phantom spectrum, and the fluorophore in a non-turbid phantom containing Bodipy FL C16 at 200 nM, no-scatter phantom spectrum.

To confirm that TMRE and Bodipy FL C16 do not exhibit optical crosstalk, four sets of phantoms were constructed containing either (i) Bodipy FL C16 at concentrations 0, 200, 400, 600, 800, and 1000 nM, (ii) TMRE at concentrations 0, 3, 6, 9, 12, and 15 nM, (iii) Bodipy FL C16 at the same concentration range with a fixed concentration of 12 nM TMRE, or (iv) TMRE at the same concentration range with a fixed concentration of 200 nM Bodipy FL C16. These phantoms contained fluorophore and polystyrene microspheres as the scatterer. No absorber was included. All concentrations were chosen to be in the biologically relevant range and within the dynamic range of the optical spectroscopy system.

In the final phantom study, a set of phantoms were created to emulate tissue optical properties. Each of the phantoms had different absorber and fluorophore concentrations and scatterer densities over a range typically found in breast tissues. The purpose of this phantom study was to demonstrate that reflectance and fluorescence measurements made at the same time with a single instrument can quantify all four endpoints: fluorescence intensities of Bodipy FL C16 and TRME, [Hb], and SO_2_. A series of ten phantoms were constructed as shown in Table 1. The diffuse reflectance from each phantom with TMRE fluorescence at 555 nm and Bodipy FL C16 fluorescence at 488 nm was measured.

### 4.6. In Vivo Murine Breast Cancer Model Studies

Three sets of animal experiments were performed to validate and demonstrate simultaneous Bodipy FL C16 and TMRE measurements. The first study aimed to demonstrate that simultaneous measurements of Bodipy FL C16 and TMRE produced equivalent results as measurements of either Bodipy FL C16 or TMRE alone in murine 4T1 solid tumors. The second aimed to analyze longitudinal changes in normal mammary tissue metabolism over an 8-week period. The third analyzed two tumor lines (4T1 and 67NR) with different metastatic potentials over a 1-week period. Seven- to fifteen-week-old female BALB/c mice (Charles River Laboratories, Raleigh, NC, USA) weighing 25 to 30 g were used in all animal studies. Animals were housed in an onsite facility with ad libitum access to food and water and standard 12-h light/dark cycles. All animal experiments were conducted during the day, and mice were fasted for at least two hours prior to optical measurements. Fasting ensured glucose in the body did not compete with metabolic probe uptake and good signal contrast from the tumor compared to normal tissue [35]. Fasting was confirmed by measuring blood glucose levels with glucose test strips (Abbott, Alameda, CA, USA). To collect reflectance and fluorescence spectra from animals, the probe was pushed gently to contact the tumor or healthy mammary fat pad, without compressing the surface or leaving an air gap, and stabilized in the same place for the duration of imaging (up to 60 min) using a custom holder. Animals were anesthetized via isoflurane breathing (1.5% isoflurane gas mixed with oxygen) throughout the course of the optical measurements.

For tumor studies, either 4T1 or 67NR murine breast cancer cell lines were used to grow orthotopic mammary tumors. The 4T1 cells were acquired from the American Type Culture Collection, and the 67NR cells were generously provided by Dr. Fred Miller (Karmanos Cancer Institute, Detroit, MI, USA) through Dr. Inna Serganova and Dr. Jason Koucher (Memorial Sloan Kettering Cancer Center, New York, NY, USA). At five weeks of age, each mouse received a 100 μL subcutaneous injection of 30,000 cells in the fourth right mammary fat pad. Tumors were monitored every other day and allowed to grow to a volume (0.5 × Length × Width^2^) of 125 mm^3^, a volume that is palpable and provides a sufficient surface for spectroscopy, without resulting in ulcerations. Additionally, a 125 mm^3^ tumor volume also allows our mice to be age-matched to our normal mammary cohort at an age of seven-eight weeks. For biological crosstalk studies, a total of twenty-seven 4T1-tumor-bearing animals were divided into three groups of nine. One cohort (*n* = 9) was injected with Bodipy FL C16 (200 μM), another cohort (*n* = 9) was injected with TMRE (75 μM), and the final cohort (*n* = 9) was injected with a mixed preparation of TMRE and Bodipy FL C16 (75 μM TMRE and 200 μM of Bodipy FL C16). Cages were randomized to minimize batch effects. All fluorophores were diluted in PBS. For longitudinal studies, a total of fifteen animals were divided into three groups of five each. One cohort (*n* = 5) received orthotopic 4T1 tumor cells, another cohort (*n* = 5) received orthotopic 67NR tumor cells, and the final cohort did not receive any tumor cells. Each cohort was injected with a mixed preparation of TMRE and Bodipy FL C16 (75 μM TMRE and 200 μM of Bodipy FL C16). All injection volumes were 100 μL.

The fluorophores were injected retro-orbitally and measurements were obtained at 2, 4, 6, 8, 10, 20, 30, 40, 50, and 60 min post-injection to quantify probe delivery. At each time point, three measurements were collected: TMRE fluorescence at 555 nm excitation and 585 nm emission, Bodipy FL C16 fluorescence at 488 nm excitation and 510 nm emission, and diffuse reflectance spectra. Bodipy FL C16 and TMRE signals at 60 min post-injection were used in all analyses [35] unless noted otherwise. The inverse Monte Carlo algorithm extracted intrinsic Bodipy FL C16 fluorescence; intrinsic TMRE fluorescence; scattering (μ_s_′) absorption coefficients (μ_a_’), and SO_2_ and ([Hb]) from μ_a_.

Fluorescence and reflectance measurements were measured over 7–15 weeks to determine any changes in the metabolic profile of normal tissue which might be impacted by animal age. Spectra were measured at 7, 8, 9, 10, and 15 weeks of age. Normal mammary tissue measurements were performed on the fourth mammary fat pad of seven- to fifteen-week-old female BALB/c mice, using the nipple as a landmark to consistently collect measurements from approximately the same anatomical location on the fat pad. For each timepoint, *n* = 5 animals were used, except at week 8 where one animal was determined to be a statistical outlier using a Grubbs’ test and at week 15, where one animal passed away. We also wanted to demonstrate that simultaneous measurements of Bodipy FL C16 and TMRE are useful in studying metabolic dependence in murine breast tumors of different metastatic potential. Fluorescence and reflectance spectroscopy was performed on each animal at 4-day intervals to collect three measurements from each tumor over the course of tumor growth (animals were 7 weeks of age by end of the study).

### 4.7. Data Analysis

All in vivo optical spectral data were processed using our previously developed scalable inverse Monte Carlo model [38,39]. The Monte Carlo model analyzed TMRE and Bodipy FL C16 fluorescence (without absorption and scattering distortions) at different time points and was used to create kinetic uptake curves. Measurement at the 60-min time point was used to quantify TMRE and Bodipy FL C16 uptake. Statistical differences between uptake curves were tested with a repeated measures analysis of variance (ANOVA). The fluorescence intensities, SO_2_, and [Hb] among different animal groups were compared using a Wilcoxon rank-sum test. A *p*-value of 0.05 or less was considered statistically significant. Pearson’s correlation coefficients and *p*-values were calculated to assess the relationship between variables. Principal component analysis (PCA) was performed on extracted metabolic measurements. The output of PCA was clustered using a built-in spectral clustering algorithm (MATLAB 2021) for both longitudinal mammary and tumor tissue data. Three separate PCAs were performed for each group of interest (mammary by age, 4T1 vs. mammary, and 67NR vs. mammary) and all principal components were included for clustering. Graphs generated show the clustering on the first two dimensions (a 2D projection onto PC 1 and PC 2 of clustering performed in 3D space on all three PCs). To quantitate the appropriateness of clustering, we calculated silhouette scores for each point using a MATLAB built-in function. Silhouette scores are calculated by the following equation:

(1) $$S\left(i\right)=\frac{b\left(i\right)-a\left(i\right)}{\max\{a\left(i\right),\;b(i)\}}$$

where *a*(*i*) is the average distance between each point and other points in their own cluster, and *b*(*i*) is the average distance between each point and the points in the opposite cluster. We calculated the mean silhouette score for each cluster and each true data label and plotted the silhouette score for each point in a silhouette plot. We also performed an information gain calculation to determine the relative information contribution of each individual input. Information gain is calculated as the difference between the Shannon’s entropy of the entire dataset and the average Shannon’s entropy for each class within the dataset. Information gain was calculated using open-source data analysis software R Studio CORElearn Library (R version 4.1.2). MATLAB (Mathworks, Natick, MA, USA) was used to perform all statistical analysis and calculations unless otherwise noted.

## 5. Conclusions

The goal of this work was to develop a non-invasive method to simultaneously measure exogenous reporters of fatty acid uptake and metabolic membrane potential and endogenous contrast related to hemoglobin concentration and tissue oxygenation. We demonstrated the feasibility of this method by showing that Bodipy FL C16 and TMRE do not crosstalk chemically, optically, or biologically, and that these probes are compatible with an inverse Monte Carlo algorithm to extract tissue optical properties and intrinsic fluorescent signal. In vivo optical spectroscopy measurements of our simultaneously-injected fluorophores revealed decreases in both TMRE uptake and total hemoglobin concentration in the mammary fat pads of healthy mice over the course of fifteen weeks. This provides new insights into how the age of mammary tissue affects the interpretation of studies comparing tumor and healthy mammary tissue. We further observed that combinations of metabolic and vascular endpoints effectively delineated clusters of normal and tumor tissues where individual endpoints could not. These observations underscore the importance of examining the relationship between the metabolic and vascular variables instead of looking at individual endpoints in isolation. This work has broad implications in tracking metabolism in vivo to study disease progression and therapy resistance.

## Figures and Tables

**Table 1:** A series of ten tissue-mimicking liquid phantoms containing Bodipy FL C16, TMRE, hemoglobin, and microspheres at varying concentrations were prepared. Table 1 shows the absorption coefficient (μ_a_, cm^−1^), reduced scattering coefficient (μ_s_′, cm^−1^), hemoglobin concentration (μM), and fluorophore concentrations (nM) for each phantom.

Phantom	μ_a_^1^ (cm^−1^)	μ_s_′ ^2^ (cm^−1^)	[Hb] (μM)	[Bodipy] (nM)	[TMRE] (nM)
1	0.14	16.72	15.09	1000	15
2	0.23	13.93	25.15	833.33	12.5
3	0.29	11.94	32.33	714.29	10.71
4	0.34	10.45	37.72	625	9.38
5	0.38	9.29	41.91	555.56	8.33
6	0.41	8.36	45.26	500	7.5
7	0.44	7.60	48.01	454.55	6.82
8	0.46	6.97	50.29	416.67	6.25
9	0.48	6.43	52.23	384.62	5.77
10	0.49	5.97	53.89	357.14	5.36

^1^ Mean absorption coefficient. ^2^ Mean reduced scattering coefficient.

## Data Availability

All data presented in this study are available in this article and in the Supplementary Material.

## Supplementary Materials

The following supporting information can be downloaded at: https://www.mdpi.com/article/10.3390/metabo12050369/s1. Supplementary Figure S1. Bodipy FL C16 and TMRE spectroscopy measurements reveal metabolic and vascular changes in the normal mammary gland, 4T1 tumor, and 67NR tumor at 7 weeks of age. Supplementary Figure S2. Inverse Monte Carlo corrected spectra collected from normal mammary tissue. Supplementary Figure S3. Inverse Monte Carlo corrected spectra collected from 4T1 tumor tissue. Supplementary Figure S4. Inverse Monte Carlo corrected spectra collected from 67NR tumor tissue.
