# Supplementary material for: A Spectroscopic Technique to Simultaneously Characterize Fatty Acid Uptake, Mitochondrial Activity, Vascularity, and Oxygen Saturation for Longitudinal Studies In Vivo
Source: Metabolites. 2022 Apr 19;12(5):369. doi: 10.3390/metabo12050369 (PMC9143017; doi:10.3390/metabo12050369)
Supplement: Supplementary file 1 [file metabolites-12-00369-s001.zip › metabolites-1680888-supplementary.pdf]

Supplementary Material

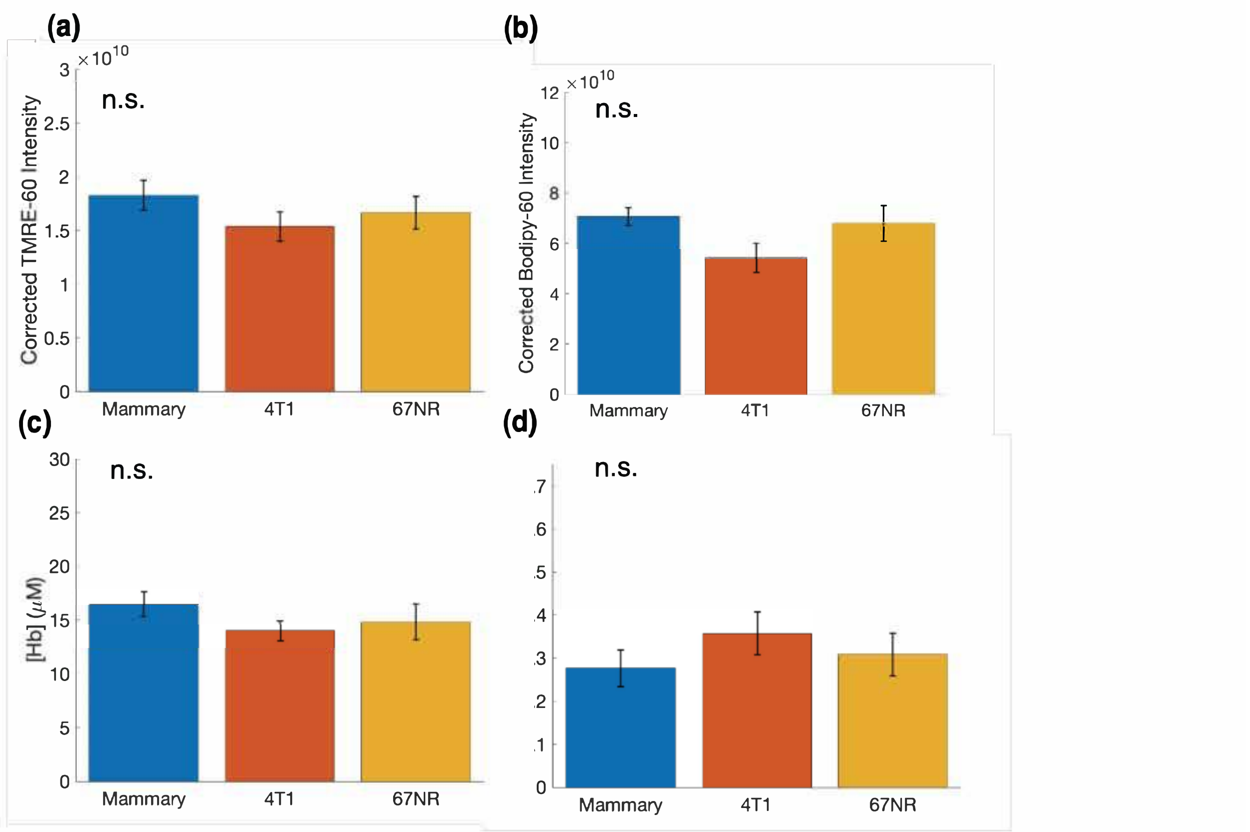

Supplementary Figure S1. Bodipy FL C16 and TMRE spectroscopy measurements reveal metabolic and vascular changes in normal mammary gland, 4T1 tumor, and 67NR tumor at 7 weeks of age. (a) Comparison of mean TMRE fluorescence intensity for different tissue types (b) Comparison of mean Bodipy FL C16 fluorescence intensity for different tissue types (c) Comparison of total hemoglobin concentration ([Hb]) for different tissue type. (d) Comparison of oxygen saturation ($[SO_2]$) for different tissue type. Error bars represent standard error (SE) of the mean along each axis. For mammary data n=9, for 4T1 and 67NR n=10. Statistical analysis was performed using a Wilcoxon rank-sum test to compare means. * is p<0.05 for all comparisons.

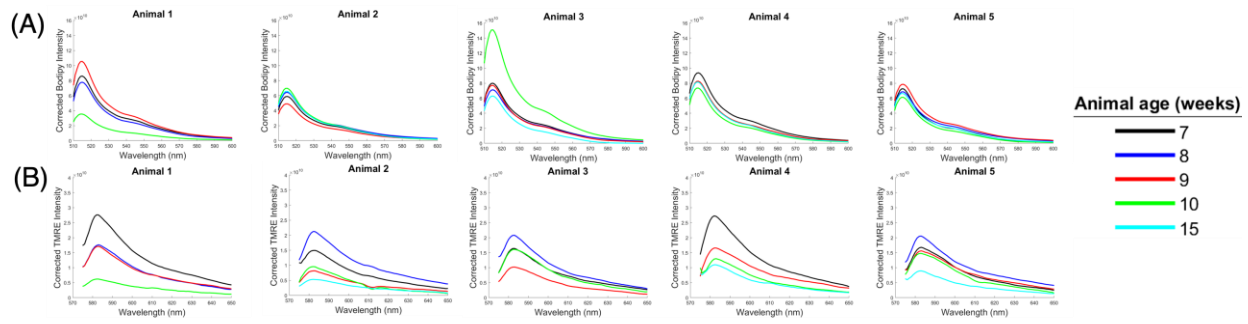

Supplementary Figure S2. Inverse Monte Carlo corrected spectra collected from normal mammary tissue. (a) Corrected Bodipy FL C16 spectra from each animal at 7, 8, 9, 10, and 15 weeks of age. (b) Corrected TMRE spectra from each animal at 7, 8, 9, 10, and 15 weeks of age. Raw spectra may be provided upon request.

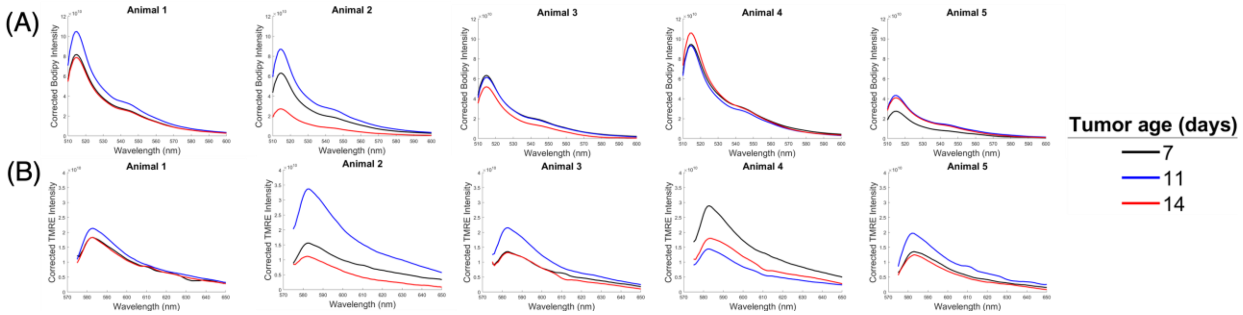

Supplementary Figure S3. Inverse Monte Carlo corrected spectra collected from 4T1 tumor tissue. (a) Corrected Bodipy FL C16 spectra from each animal at 7, 7.5, and 8 weeks of age (7, 11, and 14 days post tumor injection, respectively). (b) Corrected TMRE spectra from each animal at 7, 7.5, and 8 weeks of age (7, 11, and 14 days post tumor injection, respectively). Raw spectra may be provided upon request.

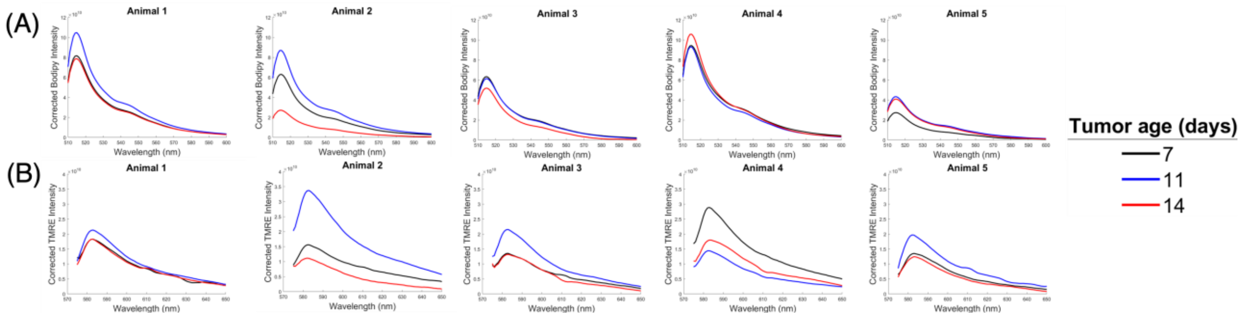

Supplementary Figure S4. Inverse Monte Carlo corrected spectra collected from 67NR tumor tissue. (a) Corrected Bodipy FL C16 spectra from each animal at 7, 7.5, and 8 weeks of age (7, 11, and 14 days post tumor injection, respectively). (b) Corrected TMRE spectra from each animal at 7, 7.5, and 8 weeks of age (7, 11, and 14 days post tumor injection, respectively). Raw spectra may be provided upon request.
